# Deterrent Effects of Essential Oils on Spotted-Wing Drosophila (*Drosophila suzukii*): Implications for Organic Management in Berry Crops

**DOI:** 10.3390/insects11080536

**Published:** 2020-08-15

**Authors:** Matthew Gullickson, Claire Flavin Hodge, Adrian Hegeman, Mary Rogers

**Affiliations:** Horticultural Science Department, University of Minnesota, 1970 Folwell Ave, Saint Paul, MN 55108, USA; gulli139@umn.edu (M.G.); flavi010@umn.edu (C.F.H.); hegem007@umn.edu (A.H.)

**Keywords:** integrated pest management, *Rubus idaeus*, small fruit, *Vaccinium corymbosum*, volatile organic compounds

## Abstract

**Simple Summary:**

Spotted-wing drosophila (*Drosophila suzukii* Matsumura; SWD) poses a significant threat to small fruit production world-wide. Though frequent applications of insecticides is the dominant strategy to manage this pest, insecticide resistance is a concern. Resistance has already been reported for one of the only consistently effective insecticides labeled for organic production systems, spinosad, underscoring the need to diversify management strategies. Botanical products, such as essential oils, contain volatile organic compounds (VOCs) which could interfere with SWD preference for or ability to locate host fruit. We conducted laboratory and field studies to determine the efficacy of botanical products (lavender oil, catnip oil, KeyPlex Ecotrol^®^ PLUS, and KeyPlex Sporan^®^ EC^2^) on preventing SWD infestation in raspberry and blueberry crops. Under laboratory conditions lavender oil, Ecotrol, and Sporan deterred SWD from diet. In the field trials, Ecotrol deterred SWD from raspberries; however, no differences were seen in blueberry infestation. To optimize essential oil deterrents for SWD, such as how to maintain effective concentrations for longer periods of time, further research is needed. Botanical deterrents represent a promising alternative pest management strategy that could be implemented without additional equipment investment from growers, while decreasing the use of broad-spectrum insecticides.

**Abstract:**

Due to concerns about frequent applications of spinosad and other broad spectrum insecticides for managing spotted-wing drosophila (*Drosophila suzukii* Matsumura, SWD), we investigated the use of essential oils as an alternative to current insecticides. Essential oils from a number of plant species have been studied for their attraction and deterrence of SWD. However, these botanical products have not been thoroughly tested in the field. We conducted laboratory and field studies to determine the efficacy of botanical products, including lavender (*Lavandula angustifolia* Mill.) oil, catnip (*Nepeta cataria* L.) oil, KeyPlex Ecotrol^®^ PLUS, and KeyPlex Sporan^®^ EC^2^ on preventing SWD infestation in raspberry (*Rubus idaeus* L.) and blueberry (*Vaccinium* *corymbosum* L.) crops. In a two-choice laboratory bioassay, lavender oil, Ecotrol, and Sporan treatments deterred SWD from a yeast-cornmeal-sugar based fly diet. In the field trials, raspberry fruit treated with Ecotrol had lower SWD infestation (6%), compared to the control (17%), and was comparable to spinosad (6%). No differences were seen in blueberry infestation. The combination of essential oils in Ecotrol may work to decrease SWD fruit infestation under certain conditions in the field, however more research is needed on the longevity of these products.

## 1. Introduction

Spotted-wing drosophila (*Drosophila suzukii* Matsumura; SWD) is an insect pest that causes devastating amounts of damage to small fruit annually [1]. SWDs have a high reproductive capacity [2], are polyphagous on soft-skinned fruit [3,4], and there is no economic threshold for damage in fresh fruit, making this pest particularly challenging for growers to manage. Unlike other species of vinegar flies, SWDs oviposit in ripening and ripe fruit instead of overripe fruit [5,6]. SWDs use visual [7] and olfactory cues to find fruit hosts [6,8,9]. Adult female SWDs can lay up to 350 eggs over their lifetime that grow from egg to adult in 11 days [2,10]. While adults feed on the exterior of the fruit, larvae feed on and develop inside of fruit [11], destroying the crop before it can be harvested [12,13].

Despite advances in integrated pest management practices [14,15,16,17], season-long broad-spectrum insecticide application remains the most prevalent management strategy for SWD [18,19]. However, there are numerous concerns associated with chemical control such as impacts on beneficial insects [20], limited options [18,21] and insecticide resistance [22]. Resistance has already been reported for spinosad [22], the only consistently effective insecticide labeled for organic production systems against SWD, underscoring the need to diversify management strategies.

A possible organic alternative to widespread broad-spectrum synthetic chemical control is the implementation of agents derived from botanical materials (e.g., essential oils, or other extracts) as insecticides, deterrents, or coupled with an attractant to develop push–pull management strategies. Push–pull techniques for agricultural pest management rely on the combination of an aversive stimulus within the primary crop, with an attractive stimulus outside of the crop to deter the pest [23]. Botanical extracts are composed of plant secondary metabolites that produce for a variety of biological functions, including disease and pest defense [24]. Plant-based essential oils may be a rich source for insect control agents given that they are principally composed of volatile organic compounds (VOCs), many of which are already perceived by insects as feeding cues, pheromones, or alarm signals [25]. Essential oils have been investigated for the management of arthropod pests such as mosquitoes (*Anopheles* spp.) [26], mites (*Tetranychus urticae* Koch) [27], swede midge (*Contarinia nasturtii* Keiffer) [28], and SWD [29,30,31,32,33,34,35].

Essential oils have been primarily used for improving baited monitoring traps for detecting adult SWDs [36,37] and to identify key attractive fruit volatiles [38] rather than as a pest reduction strategy [31,39,40]. However, botanical products may also have repellent effects on SWDs. Previous studies have shown that SWDs exhibit aversion to geosmin [9], peppermint (*Mentha × piperita* L.) oil [31,41], lavender (*Lanvandula latifolia* L.) oil [32], thymol [41] and several other essential oils or essential oil compounds [39]. Lavender oil and one of its constituent molecules, linalool, function as fumigant insecticides for SWDs at 50% effective concentration (EC50) 3.79 µL/L and 1.85 µL/L air, respectively; while 1,8-cineole (EC50 0.67%) and lavender oil (EC50 0.69%) function as contact insecticides [32]. It is hypothesized that iridoid compounds derived from catnip (*Nepeta* spp.) and kiwifruit (*Actinidia* spp.) may be repellent against SWDs due to close resemblance to VOCs produced by the parasitoid wasp *Leptopilina boulardi* Nordlander; and oviposition avoidance was observed, particularly in extracts from *Actinidia* spp. [35]. Methyl anthranilate is a VOC that occurs naturally in woodland strawberry (*Fragaria vesca* L.), and cultivars that produce high levels of methyl anthranilate resulting in decreased larval development and egg viability in SWDs, while simultaneously increasing female oviposition preference [42]. Identifying effective volatile chemical attractants and repellents for SWDs can contribute to more effective push–pull management strategies for this pest and decrease applications of broad-spectrum insecticides [40].

Commercially available blends of essential oils may help reduce damage from arthropod pests. A previous study on organic certified essential oil-based products Ecotrol and Sporan reported up to a 52% reduction in greenhouse spider mites (*Tetranyclms urticae* Koch) on tomatoes (*Lycoperscion esculentum* Mill.) after a single application [27]. Additionally, no mortality was observed on beneficial predatory mite (*Phytoseiulus persimilis* Athias-Henriot) at the label rate [27]. Ecotrol has been reported as ineffective against SWD in California field trials [12]. However, there have been no published studies on the efficacy of these commercial essential oil-based products on SWD mortality or sublethal effects such as larval development or host feeding deterrence.

Botanical products represent a promising alternative pest management strategy that could be implemented in organic production systems without additional equipment investment from growers. In addition, botanical products could decrease the organic industry’s dependence on spinosad and reduce the risk of pesticide resistance in SWD management programs. We conducted one laboratory and two field experiments to determine the effectiveness of individual oils: lavender oil and catnip oil, and the two commercially available blends of KeyPlex Ecotrol^®^ PLUS (rosemary, geraniol, and peppermint oils) and KeyPlex Sporan^®^ EC^2^ (rosemary, clove, thyme, and peppermint oils) as repellents for SWD. The objectives were to determine whether these botanical products prevent SWD oviposition and fruit infestation in the lab and field.

## 2. Materials and Methods

### 2.1. Flies

All flies were sourced from a *D. suzukii* colony reared from wild populations collected from primocane raspberries (*Rubus idaeus* L. cv. “Caroline”) in St. Paul, MN, USA (44°59′26.5″ N 93°10′27.5″ W) and supplemented annually with new wild flies. SWDs were maintained in a growth chamber, at 25 °C, 16:8 h (light:dark) photoperiod, and 47% relative humidity. SWDs were reared on an agar, yeast, sugar, and cornmeal diet medium with coffee filter paper (Essential Everyday™, Eden Prairie, MN, USA) in narrow polystyrene vials (Genesee Scientific, San Diego, CA, USA) with narrow vial Droso-plugs^®^ (Genesee Scientific, San Diego, CA, USA). Rearing methods and the diet recipe were modified from Dalton et al. [43].

### 2.2. Two-Choice Bioassay

The two-choice bioassay experiment was conducted in St. Paul, MN, during spring 2019. Based on previous research, experimental treatments consisted of lavender oil, catnip oil, and the commercially available Ecotrol and Sporan products (Table 1), in addition to a control treatment with two unscented vials (narrow drosophila polystyrene vials, Genesee Scientific, San Diego, CA, USA). The catnip oil was generated in-house by steam distillation of dried *Nepeta cataria* L. flower/seed heads collected from wild populations growing on the St. Paul campus using a 2 L Pyrex laboratory-grade still. The oil, which was denser than water, was removed and dried over anhydrous magnesium sulfate and stored in a brown glass vial. GC-MS analysis indicated that the oil was composed of ~95% nepetalactone (Figure S1). Vials were placed in bioassay arenas and were replicated 12 times for each treatment.

**Figure 1:**
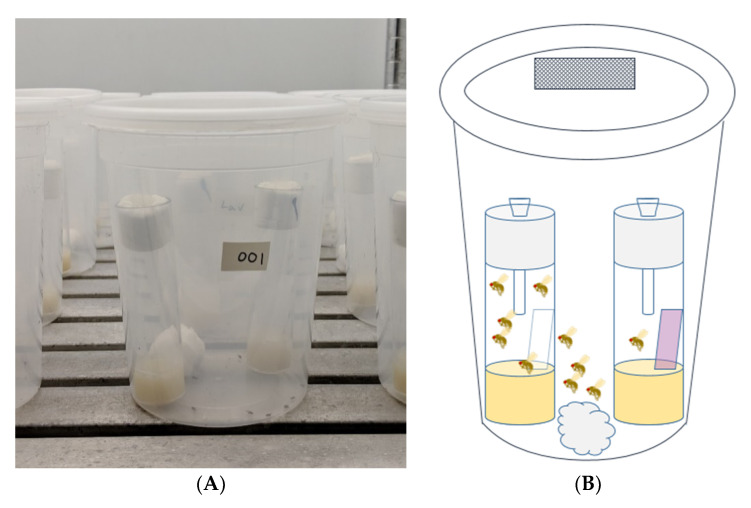
(**A**) Image of two-choice bioassay containers. (**B**) Schematic of two-choice bioassay arena constructed out of a 1 L clear plastic deli container and lid with a 1 × 2 cm section replaced with 80 g mesh netting, a moistened cotton ball, and two vials containing 5 mL of diet, a pipette-tip funnel through a foam stopper, and filter paper that was either treated with a botanical volatile organic compound or left untreated. Five male and five female *Drosophila suzukii* were placed in each container in a growth chamber for 24 h and afterwards, the number of flies in each vial was counted.

Two-choice bioassay arenas were constructed out of 1 L clear plastic deli containers with lids (Choice Paper Company, Brooklyn, New York, NY, USA) (Figure 1). Rectangular openings (1 × 2 cm) were cut in the lids and replaced with ExcludeNet^®^ (Berry Protection Solutions, Stephentown, NY, USA) 80 g insect netting to allow for gas exchange. Two polystyrene vials (Genesee Scientific, San Diego, CA, USA) filled with 5 mL of SWD fly diet (Dalton et al., 2011) were placed inside of the larger container. A 0.25 × 4 cm untreated Kimtech^®^ wipe (Kimberly Clark Corps., Minneapolis, MN, USA) was placed in one diet vial, and a 0.25 × 4 cm Kimtech^®^ wipe containing 12.5 µL of essential oil treatment was placed in the other diet vial. Treated vials were labeled underneath the vial so as not to create confounding visual stimuli. To allow the flies to enter the vials but not escape, funnels were constructed by piercing narrow vial Droso-plugs^®^ (Genesee Scientific, San Diego, CA, USA) foam stoppers with a 1 mL plastic pipette tip and then cutting ~1 cm off of the narrow end to make it wide enough for SWDs to pass through. Additionally, a cotton ball saturated with deionized (DI) water was placed in the deli container for adequate moisture. Five male and five female, 3 to 5 days old, mated flies were added to each 1 L deli container before the containers with vials and flies were placed in the growth chamber at 25 °C, with a 16:8 h (light:dark) photoperiod, and a 47% relative humidity for 24 h. We considered males in our study because, although they do not oviposit, they still make feeding decisions based on olfactory stimuli, which may be affected by the essential oil treatments. The number of flies in the untreated and treated vials was recorded after 24 h.

### 2.3. Field Experiments

The blueberry field trial took place on a commercial blueberry farm in Forest Lake, MN, USA (45°13′44.2″ N, 92°53′27.9″ W) in 2019. Half-high blueberry (*Vaccinium corymbosum* cv. “Chippewa”) plants were established in 2015. Three potted plants of the same variety were added to each tunnel to increase fruit for sampling. Plants were grown in exclusion tunnels measuring 3.6 × 2.4 × 2.1 m and were constructed by following the design used by Rogers et al. [44]. Tunnels were covered with two different plastic treatments to determine potential microclimate effects. We used a grower standard 6-mm clear poly (Polar Plastics, LLC, Oakdale, MN, USA) and Kool Lite Plus (Klerk’s Hyplast Inc., Chester, SC, USA), closed at the end walls with 80 g mesh netting (ExcludeNet^®^, Berry Protection Solutions, Stephentown, NY, USA). Tunnel plots were replicated twelve times in the allotted single row of research space at the experimental site. Each tunnel represented one replication and contained six plants (three in-ground and three potted). We randomly assigned each of the in-ground and potted plants to one of the three treatments—a total of two plants per treatment per tunnel in a random block experimental design.

Spraying was initiated on June 20, four days before the first harvest. Spray treatments were applied weekly (approximately every 7 days) and continued through August 1. Plants were sprayed at the maximum label rate (Table 1). Spray treatments consisted of a water control, Ecotrol (rosemary, geraniol, peppermint oils), and Sporan (rosemary, clove, thyme, peppermint oils), with 12 replicates of each spray treatment. Lavender oil was not used in our blueberry field trial due to plot size limitation on the commercial farm. Catnip oil was not used for the same reasons as lavender oil in addition to showing no effect in the laboratory two-choice experiment.

Directly following spraying, approximately 25 female and 25 male SWDs were released from the northwest corner into the enclosed tunnels for artificial infestation. Four days after spray application, all ripe berries were harvested. Harvest of the potted plants began on June 24, while harvest of the in-ground plants started July 15. The last harvest was completed August 5. Releasing flies from a laboratory colony into the tunnels was not sufficient to build SWD populations within the tunnel, and therefore the netting from the tunnels was removed seven days prior to the last harvest on August 5 to allow natural infestation to occur. Therefore, August 5’s data show a covered open-plot simulation. A random subsample of 10 individual berries from each spray treatment replicate was placed in 37 mL plastic containers, incubated for 7 days in a growth chamber (25 °C, 16:8 L:D photoperiod, and a 47% relative humidity), and the proportion of infested fruit was determined visually using a dissecting microscope.

The raspberry trial experiment took place at the University of Minnesota Research and Outreach Center in Rosemount, Minnesota (44°43’40.3” N, 93°05’48.8” W) in 2019. Primocane raspberry (*Rubus idaeus* cv. “Heritage”) plots were established in 2014. Plots were planted in double rows, 7.5 m long and 1.5 m wide, with 1.5 m spacing between rows; each plot consisted of two 3.75 m long treatment sections. Five treatments were replicated four times, for a total of 20 plots. Plots were arranged in a randomized split block design.

Spraying was initiated on August 27, one week prior to ripe fruit, when raspberries were at the “yellow” to “pink” fruit stage of ripeness. Spray treatments were applied every 5 to 9 days, depending on weather conditions and continued through the 5-week harvest window. Plants were sprayed at the maximum label rate (Table 1). Spray treatments consisted of an untreated control, lavender oil, Ecotrol, Sporan, and spinosad. Catnip oil was not used in the field trial because of a lack of efficacy observed in the laboratory two-choice experiment.

We used certified organic store-bought sentinel fruit to assess fruit infestation in the field to minimize the possibility that the fruit would be infested prior to spraying. It is possible that store-bought fruit is previously infested, and we assessed a sub-sample of 40 fruit and found a total of 16 eggs, or 0.4 eggs/fruit. However, none of these eggs were viable, likely due to post-harvest cold storage treatment [44]. Consequently, we measured larval infestation in fruit rather than in the number of eggs. Store-bought raspberries were briefly rinsed with DI water, dipped in a 2% propionic acid solution, and then allowed to air-dry in the laboratory to remove insecticide residues. Five store-bought berries were placed in 10 cm diameter Petri dishes and placed on the canopy floor within the raspberry canopy in the center of the treatment plots. These were set out on three dates (September 13, September 22, and October 7) during the harvest window just prior to spraying. We sprayed the store-bought fruit at the same time as the larger plots, and left fruit in the plots for 24 h. Individual berries were placed in 37 mL plastic containers, incubated for 7 days in a growth chamber (25 °C, 16:8 L:D photoperiod, and a 47% RH), and the proportion of infested fruit was determined visually under 10× magnification.

### 2.4. Data Analysis

In the two-choice experiment, the number of flies (combined totals of males and females) in the treatment vials was subtracted from the number of flies in the control vial for each replicate to obtain the difference between the two vials, similar to the oviposition index in Karageorgi et al. [5]. Differences were normally distributed, and therefore a one-way ANOVA was used to compare these differences between treatments and the control for significance at the α = 0.05 level.

In the blueberry field experiment, data were zero inflated due to low levels of infestation for all treatments until after July 29, therefore, to analyze the effects of essential oil treatment on infestation, a zero inflated negative binomial generalized linear model (GLM) was applied with the proportion of SWD infested fruit as the response variable and sprays were the experimental treatments. Plastic covering did not have a significant effect on the infestation, and therefore was pooled. The GLM was followed by analysis of deviance and pairwise chi-squared tests for multiple comparisons and a post hoc Tukey’s mean separation test of SWD fruit infestation for significance at the α = 0.05 level.

For the raspberry field trial, the proportion of raspberries infested with SWD was heavily skewed and therefore was arcsine transformed. Untransformed means and the standard error of the mean (SEM) are reported. Data were analyzed using a one-way ANOVA and a post hoc Tukey’s mean separation test for significance at the α = 0.05 level. Data for proportion infestation were the response variable and the products tested were the treatments. Data were analyzed using R [45].

## 3. Results

### 3.1. Two-Choice Laboratory Experiment

**Figure 2:**
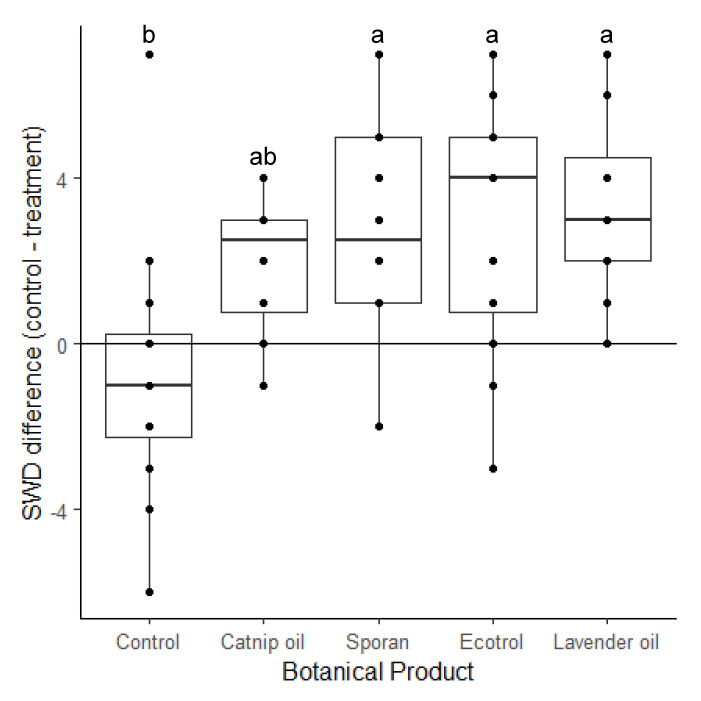
The difference in the number of *D. suzukii* (spotted-wing drosophila, SWD) between the untreated vials minus the number of SWD in the treated vials. The control treatment is the difference between two untreated vials. Data points above 0.00 indicate a greater number of flies in the untreated vials than the treated vials. Letters denote significant differences at the α = 0.05 level (F = 11.49, df = 5, 55, *p* < 0.001).

SWDs preferred not to select diet vials that were treated with botanical products (F = 11.49, df = 5, 55, *p* < 0.001). The difference in number of flies between the two vials (mean ± SEM, *p*-value) for the control treatment (0.83 ± 0.94) was not significantly different from zero, and this difference was significantly smaller compared to the lavender (−3.33 ± 0.62, *p* = 0.002), Ecotrol (−2.92 ± 0.89, *p* = 0.006), and Sporan (−2.83 ± 0.72, *p* = 0.008) botanical product treatments, however, there was not a significant difference between the catnip treatment and control (−1.92 ± 0.45, *p* = 0.08). Essential oils deterred SWD in the laboratory, with SWD preferring to enter the untreated vial more often than the scented treatment vials (Figure 2). There were no differences between the four botanical product treatments.

### 3.2. Blueberry and Raspberry Field Experiments

In the blueberry field experiment, the spray treatments (χ^2^ = 0.191, df = 2, *p* = 0.909) did not have an effect on the proportion of SWD infested fruit. Estimated blueberry infestation from the zero inflated model is reported in Table 2.

In the raspberry field experiment, the proportion of SWD infested fruit (mean ± SEM) was significantly different between treatments (F = 6.19, df = 4, 53, *p* < 0.001). Ecotrol (0.06 ± 0.01) and spinosad (0.06 ± 0.02) both had significantly lower levels of infestation compared to the unsprayed control (0.17 ± 0.04). The lavender oil and Sporan treatments did not have lower levels of SWD infestation compared to the unsprayed control (Table 3).

## 4. Discussion

This research adds to the growing body of evidence that certain botanical products could provide an alternative pest management strategy for SWD that could be organically implemented and further developed into effective push–pull management techniques.

When presented with a choice of oviposition and feeding substrates, SWDs had a greater preference for the untreated diet vials compared to the lavender oil, Ecotrol, or Sporan treated vials (Figure 2). Keesey et al. [35] also observed decreased oviposition on substrates treated with *Actinidia* spp. essential oils, but not for catnip treated substrates. These results suggest that aversion, rather than mortality could be the reason for any observed lower infestation rates in the field. Our results that specific volatile essential oils can be aversive to SWDs are consistent with other laboratory and field studies [32,41]. Previous laboratory studies have also noted that certain essential oils have stronger effects than others [35,41], however, we did not observe any differences under laboratory conditions.

Previous laboratory studies on using essential oils as a pest management strategy for SWDs have shown that lavender oil, avocado oil, peppermint oil, and thymol are effective at preventing SWDs from landing on treated surfaces, but have not been adequately tested in the field [32,41]. In our raspberry field trials, the commercially available blend of rosemary oil, geraniol, and peppermint oil had significantly lower rates of SWD fruit infestation compared to the unsprayed control in raspberries and was comparably effective to spinosad (Table 3). However, no differences were seen in blueberry infestation. The two other essential oil treatments in the raspberry experiment (the lavender oil and Sporan treatments) did not result in lower SWD fruit infestation compared to the control. A combination of rosemary oil, geraniol, and peppermint oil may be a viable essential oil blend for deterring SWDs in the field, which supports assessment from Renkema et al. [41] that a blend of essential oils may be more effective than a single oil alone. In the blueberry field experiment, the lack of observed differences between essential oil treatments could be due to the low levels of overall SWD infestation until the last two weeks of the harvest season, effectively reducing our experimental sample size. Wallingford et al. [40] coupled 1-octen-3-ol, an aversive botanical VOC to SWDs, with a baited trap to create a push–pull treatment that was more effective than either the deterrent or the attractive bait alone.

One of the challenges associated with interpreting and comparing the results from studies using essential oils or commercial preparations of these oils is that they may be fairly variable in their chemical composition from batch to batch. Given that high concentrations of oil components may be acutely toxic due to general hydrophobic chemical solvent effects such as cuticle or cellular damage, it is important to consider the composition and concentrations of individual chemicals in essential oils to tease apart different modes of action. Ideally, specific modes of action that target insect behavioral responses at low concentrations are more likely to have practical utility than general toxicity observed at higher concentrations. Several previous studies have attempted to define the activity of constituents of essential oils. One such study showed that lavender oil may be a viable fumigant for SWD due to the monoterpene components 1,8-cineole, carene, and linalool [32]. Additionally, Gowton et al. [46] found that fewer SWD pupae in the soil reached adulthood when they were treated with peppermint oil fumigation. Other botanically derived chemicals, such as methyl benzoate, may be more effective as insecticides. Feng and Zhang [34] report 100% *D. suzukii* mortality was achieved when adult flies were exposed to a 1% concentration of the botanical VOC methyl benzoate applied to blueberries. Other VOCs tested did not result in significant mortality. Feng and Zhang [34] also compared SWD mortality when exposed to the botanical VOCs (α-terpinene, γ-terpinene, α-terpineol, α-pinene, and 1,8-cineole) and found that these other VOCs did not result in increased mortality compared to the water control. One specific mode of action for essential oils is the interference with *Drosophila* octopamine receptors in the nervous system, where essential oils may function as either an agonist or antagonist to G-protein binding site [47]. The differences in results across studies could be due to differences in experimental design (e.g., arena size, VOC concentration, application method, etc.); while these oils may increase SWD mortality under certain conditions, replicating specific conditions in the field would be challenging, and therefore specific and sub-lethal effects of these oils should be investigated further.

A major hurdle to implementing botanical products for horticultural pest control is the uncertainty around how to effectively utilize volatile compounds in the field (e.g., volatilization rate, application method, and impact on infestation in small fruit) and economic feasibility. Of the few studies that have investigated essential oil deterrence in situ, methods for deploying the essential oils range from spray applications, wicking materials [40], polymer flakes [41] to intercropping with fragrant plant species [48,49]. Intercropping strawberries with peppermint plants that were periodically mowed to increase the amount of volatiles has been shown to actually increase the amount of SWD infestation in the fruit, potentially due to providing additional shaded habitat for SWDs during the day [49]. The greatest challenge to implementing an effective deterrent management tactic will be maintaining biologically relevant amounts of VOCs in the field. Wallingford et al. [30] estimate that essential oil diffusion of 10 mg/hour is enough to cause deterrence in SWDs, however, they do not state over how large of an area that this rate would cover. An important next step in this research will be to test on beneficial insects, such as pollinators, to make sure there are no negative consequences associated with applying essential oils. Wallingford et al. [40] showed that pollinator visitation rates did not decrease when essential oils were utilized in a raspberry field study. There is reasonable concern about non-target effects since plants often produce these molecules as defenses against a wide range of arthropods and other phyla [24,30]. However, these products mitigate many of the problems associated with insecticide application. Botanical products are often regarded as safe for humans and the environment [24], with a few exceptions; they have a short residual activity [27], would decrease the amount of broad-spectrum insecticides, and may decrease insecticide resistance pressures.

Additionally, one of the challenges of organic insecticide research and management is the short residual activity of many organic insecticides. Of the products we tested, spinosad has the longest preharvest interval at 1 day, whereas Ecotrol and Sporan have no preharvest interval. Positive consequences of the short residual activity may be less beneficial insect (e.g., pollinators and parasitoids) mortality and shorter re-entry and pre-harvest intervals. Negatively, the shortened residual activity of organic insecticides may not protect ripening fruit for as long as their synthetic counterparts. However, the lack of effective options for organic SWD management and documented resistance to spinosad highlight the need for additional management strategies. Therefore, we recommend further research to investigate dosage response modeling, modes of action, and application methods of novel insecticides and botanical deterrents on SWD.

## 5. Conclusions

Insecticide application is still the most widespread control method for SWD, despite recent advances in integrated pest management tactics and concerns about sustainability. Botanical volatiles are known to mediate plant–insect interactions and our results indicate that essential oils containing these compounds could be exploited to deter SWDs from feeding and ovipositing on treated fruit. Lavender oil, Ecotrol, and Sporan were aversive to SWDs in the laboratory, and Ecotrol was effective in our raspberry field trial. The utilization of botanical products may be a viable SWD organic management strategy, and therefore should be investigated further.

## Figures and Tables

**Table 1:** Essential oil treatments with the percentage of active ingredients (% AI) investigated for insecticidal or deterrent effects on *Drosophila suzukii*.

Treatment (% AI)	Trade Name	Manufacturer	Rate	Experiment(s)
*Lavandula angustifolia* (100%)	Lavender oil	Now^®^ Foods, Bloomingdale, IL, USA	19.0 mL/hectare	Two-choice and raspberry field trial
*Nepeta cataria* (100%)	Catnip oil	Steam distilled in-house	NA *	Two-choice
Rosemary (10%), geraniol (5%), and peppermint (2%) oils	Ecotrol^®^ PLUS	KeyPlex, Winter Park, FL, USA	3.5 L/hectare	Two-choice, blueberry and raspberry field trials
Rosemary (16%), clove (10%), thyme (10%), and peppermint (2%) oils	Sporan^®^ EC^2^	KeyPlex, Winter Park, FL, USA	3.5 L/hectare	Two-choice, blueberry and raspberry field trials
Spinosad (22.5%)	Entrust^®^ SC Naturalyte^®^	Corteva^TM^ Agrisciences, Indianapolis, IN, USA	0.44 L/hectare	Raspberry field trial

* *Nepeta cataria* was only tested in the two-choice experiment, and therefore does not have a field application rate.

**Table 2:** Estimated proportion of *Drosophila suzukii* infested blueberry fruit applied with two botanical volatile organic compounds and grown under two kinds of plastics in a semi-field bioassay. Spray treatments include control, Ecotrol (rosemary, geraniol, peppermint oils), and Sporan (rosemary, cloves, thyme, peppermint oils). Fruit was harvested 4 days after spraying before incubating at 24 degrees Celsius for 7 days to determine proportion of infested fruit per treatment. No significant differences were detected at the α = 0.05 level.

Spray	Estimated Proportion SWD Infested Fruit (Mean ± SEM)
Control	0.28 ± 0.07
Ecotrol	0.23 ± 0.06
Sporan	0.25 ± 0.06

**Table 3:** Treatment efficacy on the proportion of *Drosophila suzukii* infested fruit in a semi-field bioassay of primocane raspberries. Store-bought fruit was sprayed in the field and left for 24 h before incubating at 24 degrees Celsius for 7 days to determine the proportion of infested fruit per treatment. Letters denote significance at α = 0.05.

Treatment	Proportion SWD Infested Fruit (Mean ± SEM)
Unsprayed control	0.17 ± 0.04 *a*
Lavender oil	0.22 ± 0.06 *a*
Sporan	0.15 ± 0.04 *ab*
Ecotrol	0.06 ± 0.01 *b*
Spinosad	0.06 ± 0.02 *b*

## Supplementary Materials

The following are available online at https://www.mdpi.com/2075-4450/11/8/536/s1, Figure S1: GC-MS Analysis of *Nepeta cataria* Essential Oil.
