# Supplementary figures and images for: Deterrent Effects of Essential Oils on Spotted-Wing Drosophila (Drosophila suzukii): Implications for Organic Management in Berry Crops
Source: Insects. 2020 Aug 15;11(8):536. doi: 10.3390/insects11080536 (PMC7469169; doi:10.3390/insects11080536)

## GC-MS Analysis of *Nepeta cataria* Essential Oil

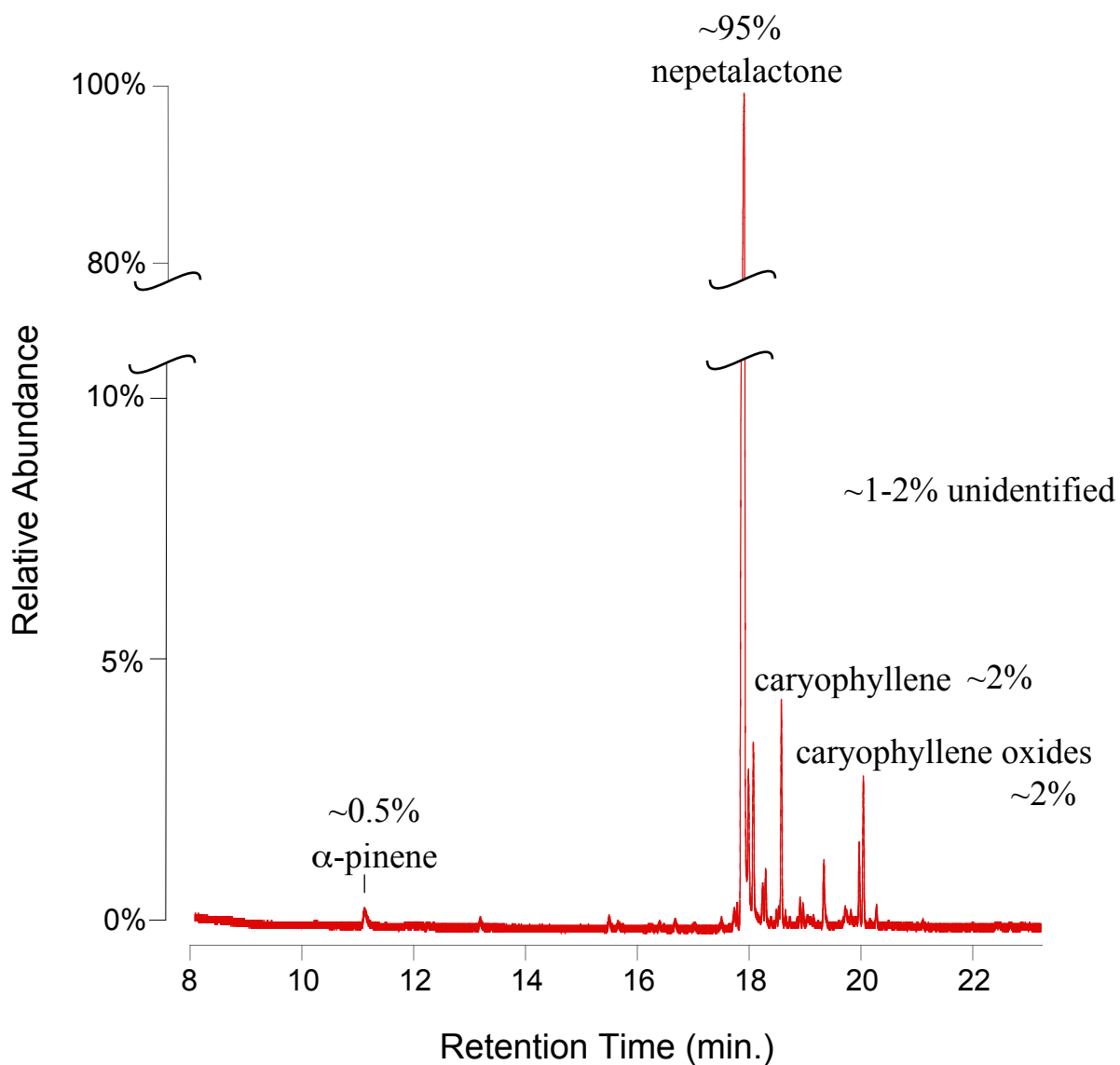

Supplement: Supplementary file 1 [file insects-11-00536-s001.zip › insects-860650-supplementary/Nepeta GC-MS.pdf]
